# The Implicit Positive and Negative Affect Test: Validity and Relationship with Cardiovascular Stress-Responses

**DOI:** 10.3389/fpsyg.2016.00425

**Published:** 2016-03-30

**Authors:** Melanie M. van der Ploeg, Jos F. Brosschot, Julian F. Thayer, Bart Verkuil

**Affiliations:** ^1^Health, Medical and Neuropsychology Unit, Institute of Psychology, Leiden UniversityLeiden, Netherlands; ^2^Department of Psychology, The Ohio State UniversityColumbus, OH, USA; ^3^Clinical Psychology Unit, Institute of Psychology, Leiden UniversityLeiden, Netherlands

**Keywords:** stress, prolonged cardiovascular activity, reactivity/recovery, harassment, unconscious stress, implicit affect, implicit measures, IPANAT

## Abstract

Self-report, i.e., explicit, measures of affect cannot fully explain the cardiovascular (CV) responses to stressors. Measuring affect beyond self-report, i.e., using implicit measures, could add to our understanding of stress-related CV activity. The Implicit Positive and Negative Affect Test (IPANAT) was administered in two studies to test its ecological validity and relation with CV responses and self-report measures of affect. In Study 1 students (*N* = 34) viewed four film clips inducing anger, happiness, fear, or no emotion, and completed the IPANAT and the Positive And Negative Affect Scale at baseline and after each clip. Implicit negative affect (INA) was higher and implicit positive affect (IPA) was lower after the anger inducing clip and vice versa after the happiness inducing clip. In Study 2 students performed a stressful math task with (*n* = 14) or without anger harassment (*n* = 15) and completed the IPANAT and a Visual Analog Scale as an explicit measure afterwards. Systolic (SBP), diastolic (DBP) blood pressure, heart rate (HR), heart rate variability (HRV), and total peripheral resistance (TPR) were recorded throughout. SBP and DBP were higher and TPR was lower in the harassment condition during the task with a prolonged effect on SBP and DBP during recovery. As expected, explicit negative affect (ENA) was higher and explicit positive affect (EPA) lower after harassment, but ENA and EPA were not related to CV activity. Although neither INA nor IPA differed between the tasks, during both tasks higher INA was related to higher SBP, lower HRV and lower TPR and to slower recovery of DBP after both tasks. Low IPA was related to slower recovery of SBP and DBP after the tasks. Implicit affect was not related to recovery of HR, HRV, and TPR. In conclusion, the IPANAT seems to respond to film clip-induced negative and positive affect and was related to CV activity during and after stressful tasks. These findings support the theory that implicitly measured affect can add to the explanation of prolonged stress-related CV responses that influence CV health.

## Introduction

Psychosocial stressors such as marital stress and job stress are increasingly recognized as contributors to the development or progress of cardiovascular (CV) disease (see for example McEwen, 1998, 2003; Rozanski et al., 1999; Rosengren et al., 2004; Strike and Steptoe, 2004; Brotman et al., 2007; Chida and Hamer, 2008; Dimsdale, 2008; Lu et al., 2013). Still, studies have been inconclusive on the mechanisms underlying the relationship between psychosocial stress and CV diseases (Dimsdale, 2008; Brindle et al., 2014). This might be related to the inability of the used measurements of psychological stress to explain CV activity (Gerin et al., 2006; Key et al., 2008; Pieper et al., 2010). The current paper addresses this issue by validating a test that indirectly assesses affect and is expected to more closely relate to psychophysiological responses; the Implicit Positive and Negative Affect Task (IPANAT; Quirin et al., 2009a; Quirin and Lane, 2012).

The reactivity hypothesis of stress has been the main focus of the field and emphasizes the acute physiological responses during a stressor. However, accumulating literature suggests that prolonged stress responses and not, or to a lesser extent, the reactivity during stressors, determine the detrimental consequences for health. In other words, measuring the CV activity during stressors might not fully represent that part of the physiological stress response that explains the development of CV or other diseases. Slow recovery from stressors and anticipatory responses to them might be of equal or even greater importance (Haynes et al., 1991; Linden et al., 1997; Ursin and Eriksen, 2004; Pieper and Brosschot, 2005; Koolhaas et al., 2011; Panaite et al., 2015). Moreover, this prolonged activity leads to a pathological state that is often described as “allostatic load” (McEwen, 1998) and is the final biological pathway to organic disease. Earlier research focusing on reactivity to a stressor has overlooked these different forms of the maladaptive stress response, i.e., prolonged physiological activation. These forms of prolonged activation have been attributed to ongoing cognitive representation of the stressors, which is known as perseverative cognition. Perseverative cognition, often manifested as rumination or worry, has been associated with prolonged CV activity (Brosschot et al., 2006, 2007; Pieper et al., 2007; Juster et al., 2012; see for reviews Verkuil et al., 2010; Ottaviani et al., 2016).

The assessment of psychological stress to explain related CV responses is typically done through self-report methods such as keeping a worry and mood diary or completing questionnaires like work stress scales or trait questionnaires of worry, anxiety, or general negative affect (e.g., Gerin et al., 2006; Brosschot et al., 2007; Pieper et al., 2007, 2010; Key et al., 2008; Verkuil et al., 2012). However, several findings indicate that these measures do not fully explain the prolonged CV responses to stressors (Gerin et al., 2006; Key et al., 2008; Pieper et al., 2010). Brosschot et al. (2007) for example found that individuals that experienced stressors and worry during the day displayed increased cardiac activity during sleeping at night, when conscious worry and affect related cognitions are absent. Moreover, Pieper et al. (2010) demonstrated that cardiac effects of worry in real life continued after worry episodes ceased and were not due to negative affect or bio-behavioral variables such as movement or smoking. Additionally, Gerin et al. (2006; Key et al., 2008) found that slow blood pressure (BP) recovery after an experimental stressor was not due to explicit worrisome thoughts. These findings seem to indicate that part of the psychological stress response affects the CV system in a way that is not addressed by self-report measures. Brosschot and colleagues (Brosschot, 2010; Brosschot et al., 2010) have hypothesized that this part is explained by ongoing unconscious (or “implicit”) stress-related cognition. This unconscious stress-related cognition would represent a general negative state that one is unable to express, but that does affect physical wellbeing. Concepts related to unconscious stress-related cognition have already been widely used within cognitive and social psychology, such as implicit affective attitudes, self-esteem, and emotion (see for example Kihlstrom, 1987; Fazio and Olson, 2003; Bargh and Morsella, 2008; Gyurak et al., 2011), and have been demonstrated to influence for example decision making processes (Dijksterhuis et al., 2006) and affective evaluation (Zajonc, 1980). Implicit stress-related cognition cannot be measured with self-report methods, because for these methods deliberate processing of the assessed construct is required (De Houwer et al., 2009).

Various instruments have been designed to measure affective processing at an implicit level, i.e., implicit measures, such as the affective Implicit Association Test (IAT; Egloff et al., 2002; Verkuil et al., 2014) and the Implicit Positive and Negative Affect Task (IPANAT; Quirin et al., 2009a). In the current study, we examined the IPANAT as an implicit measure of stress-related cognition operationalized as implicit affect (Quirin et al., 2009a). The IPANAT is suggested to operate as an implicit measure of affect through the process of affect misattribution (Zajonc, 1980; Forgas, 1995; Payne et al., 2005; Quirin and Bode, 2014). Similar to the original studies of Zajonc and colleagues in the IPANAT (1980) ambiguous stimuli are presented, namely a set of nonsense words, of which the affective value is rated on a six point scale for 12 emotional adjectives. The assumption is that the participants, again as in Zajonc's studies, respond in accordance with their current affective state, without being fully aware of the construct being measured (Quirin et al., 2009a). The implict negative affect scale (INA) of the IPANAT has been shown to predict cortisol responses to a speech stressor and increases in circadian cortisol concentrations (Quirin et al., 2009b). The latter was recently partly replicated by Mossink et al. (2015). In Brosschot et al. (2014, Study 2) INA, measured with the IPANAT, was related to slower recovery of BP after a math stressor with anger harassment, whereas explicit negative affect (ENA) showed no significant relationship. However, in that study no control group for extra negative affective changes due to harassment was used, which limits inferences on the application of the IPANAT as implicit measure of stress-related cognition. In the current study, the harassment manipulation was again tested and a control group with only a math task was added to the design to test whether it is the specific affective component of anger harassment that affects INA and IPA as measured with the IPANAT.

The present studies address two issues. First, the IPANAT's content validity has hitherto only been tested with simple affective stimuli, namely pictorial emotional stimuli. Furthermore, although associations of the IPANAT with physiological measures have been found its relationship with explicit measures of affect are underappreciated (for a review see Quirin et al., 2009a; Quirin and Bode, 2014). For example Quirin et al. (2009b) found a relationship between the negative, but not the positive, subscales of implicit and explicit affect. However, this observational study measured changes in cortisol levels, but not in affect. Thus, the interpretation of both the relationship between implicit and explicit affect and the ability of the IPANAT to capture direct changes in affect due to stressful experiences cannot readily be applied to the current ideas about unconscious stress-related cognition. In the current two studies content validity was examined under more realistic conditions by providing negative and positive emotional film clips in one study, which are more ecologically valid than simple pictures and have been suggested to elicit prolonged affective responses compared with pictures (e.g., Gross and Levenson, 1995; Rottenberg et al., 2007; Schaefer et al., 2010), and by deploying a more naturalistic stressor, namely a math task with and without anger harassment in a second study. Moreover, in the first study we assessed the IPANAT's ability to detect changes in (implicit) affect and in the second study we relate the IPANAT subscales to physiological parameters to more specifically address the theory that changes in these parameters can be related to affect measured implicitly. We expected that the emotional film clips and especially anger harassment would evoke affect-congruent changes on the IPANAT subscales that are at least partly independent of explicit affect. Second, it addresses whether CV responses during a stressor and recovery from it, as a model of prolonged CV activation, are associated with implicit affect as measured with the IPANAT and whether this association is at least partly independent of that of explicit affect. More precisely, we expected that INA would be related to a higher reactivity to a stressor and slower recovery from it, and vice versa for implicit positive affect (IPA).

Furthermore, we expected stronger affective and CV effects for the math stressor with harassment. CV recovery is typically longer after emotional stressors than after physical or neutral stressors, while reactivity (i.e., responses during these stressors) is often equally high (e.g., Brosschot et al., 2014, Study 1; Linden et al., 1997). This difference in recovery is taken to be due to prolonged explicit stress-related cognition, or high ENA or low explicit positive affect (EPA), or both. Here, we hypothesized that it is also due to implicitly measured affect, that is high INA or low IPA, or both. Consequently, we expected that a more strongly negative emotional stressor (math with harassment) would lead to slower CV recovery and higher negative and lower positive affect, measured explicitly and implicitly, than a relatively more neutral stressor (math without harassment). We also expected that the slower CV recovery after harassment would be explained by the stronger affective responses, and that implicit affect explains CV recovery over and above explicit affect.

In sum, previous findings suggest that the IPANAT might be a suitable implicit measure of stress-related affective cognition, but its content validity and its ability to explain CV activity, expressed as reactivity and recovery to an emotional stressor, have not been thoroughly examined. In the present article two studies are reported that tested whether the IPANAT is able to detect changes in affective state induced by emotional film clips (Study 1) and whether it can explain CV responses to a stressor beyond explicit measures of affect (Study 2). In addition, it was tested whether the IPANAT scores were related to the general and differential CV responses to a stressor with and without anger harassment and to CV recovery after these stressors.

## Study 1

### Methods

#### Participants and procedure

**Figure 1:**
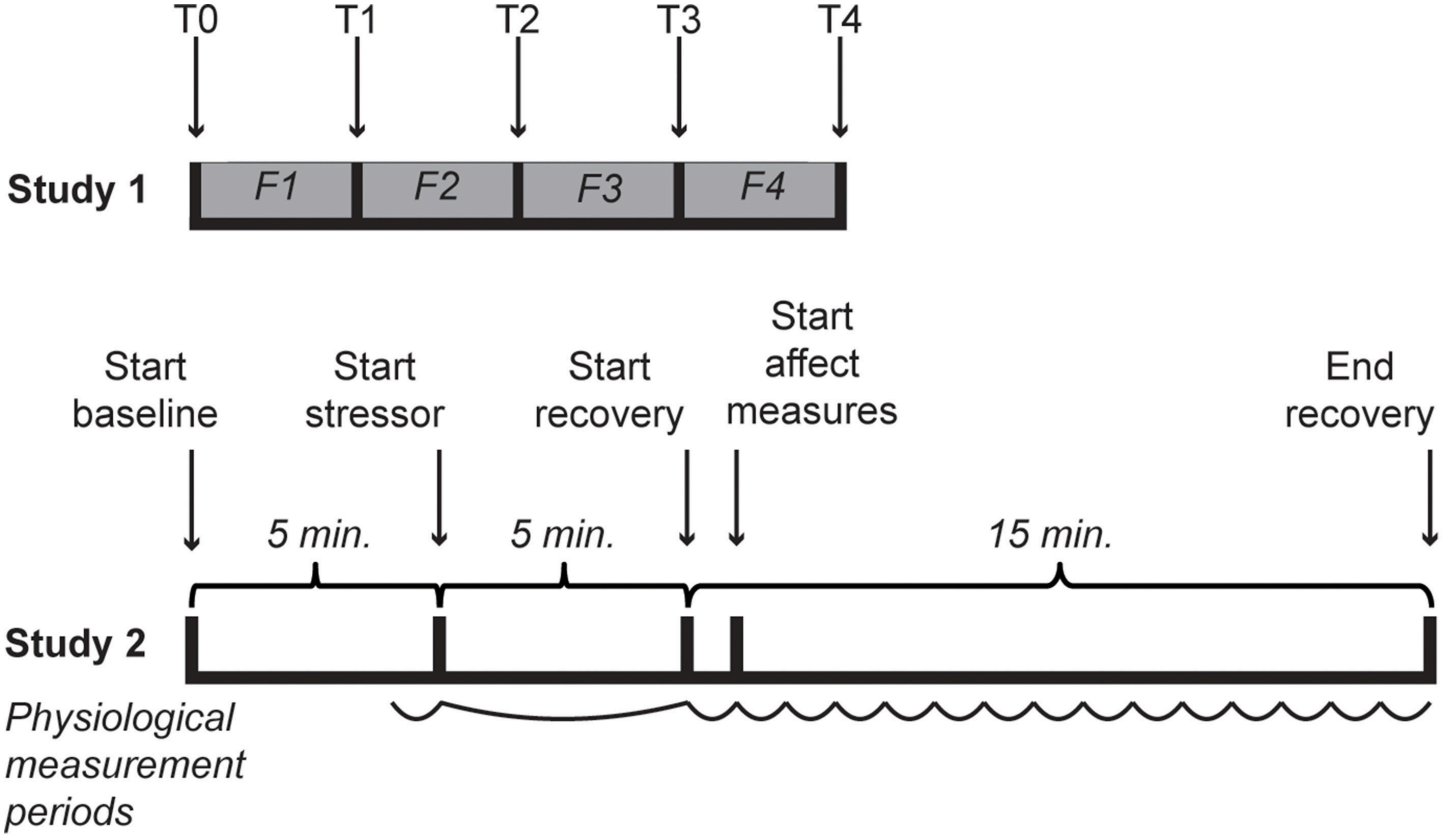
**Timelines of both studies**. In Study 1 T0 represents the baselines measurement of affect, while T1–T4 represent the affect measures after each film clip (indicated with F1–F4). During Study 2 cardiovascular activity was measured throughout. For analyses the last minute of baseline, the 5-min stressor and 15 separate min of the recovery were used, as indicated with a curved line.

A total of 34 [64.7% female; mean age of 24.0 (*SD* = 8.51)] students of Leiden University with sufficient understanding of the Dutch language enrolled in the experiment for course credits or five euro. Participants provided informed consent and received the standard instructions for the questionnaires after which they were seated in front of a computer and were asked to put on a Sennheiser HD201 headphone. In random order, four film clips were shown that were previously validated to elicit anger, happiness, fear and a neutral state. The film clips were English versions identical to code 15 (1:17 min.), 24 (2:45 min.), 65 (3:57 min.) and 55 (0:40 min.), respectively, from the FilmStim database (Schaefer et al., 2010). The volume accompanying the film fragments was set at medium (45–55 dB). The IPANAT and Positive And Negative Affect Scale (PANAS; Watson et al., 1988) were administered at baseline and after each video clip (see Figure 1). In one case the PANAS was not completed after the anger film clip. The study was approved by the Independent Ethics Committee of the Institute of Psychology of Leiden University, under number 5148415681.

#### Implicit and explicit affect

A Dutch translation of The Implicit Positive and Negative Affect Test (IPANAT) as a measure of implicit affect was provided (Quirin et al., 2009a; Brosschot et al., 2014). Respondents rated six artificial words (vikes, tunba, ronpe, belni, sukov, safme) for emotional adjectives on a six-point Likert scale. In the version we used, the IPANAT for discrete emotions (Quirin and Bode, 2014), 12 emotional adjectives are used. The mean scores per adjective for all artificial words were computed and summarized in the mean scores of INA (sad, gloomy, unhappy, annoyed, irritated, angry, afraid, frightened, scared) and IPA (joyful, cheerful, happy). In this particular study the IPANAT was used as a repeated measure by providing the entire IPANAT at baseline and two nonsense words, randomly selected from the pool of six words, after each film clip. Repeated presentation of the same full test was likely to cause carryover and training effects or boredom, resulting in erroneous scoring. Filling out the full version IPANAT takes about 5 min and as a repeated measure about 2 min for each administration. In the current sample the IPANAT administered at baseline was found to be reliable with Cronbach's α = 0.75 for INA and Cronbach's α = 0.89 for IPA, which is comparable to the reliability found by Quirin et al. (2009a).

At all measurement points explicit affect was measured with the PANAS, which measures positive and negative affect on two 10 item scales with emotional adjectives (Watson et al., 1988). Participants indicate on a five-point scale the extent to which the items apply to their current affective state. The PANAS was found reliable in this sample with Cronbach's α = 0.90 for ENA, Cronbach's α = 0.87 for explicit positive affect (EPA), which is comparable with reliability found by Crawford and Henry (2004) in a large non-clinical sample. The implicit and explicit affective responses to video clips were compared with the affective responses at baseline.

### Results

The demographical information of all participants is provided in Table 1. Mean affect scores are displayed in Table 2. In this within-subjects design, the effect of the film clips on affect was determined with four one-way repeated measures ANOVA's, one for each affect measure. There were significant differences between film clips on all affect measures [INA: Wilks' λ = 0.51, *F*_(4, 30)_ = 7.32, multivariate partial η^2^ = 0.49; IPA: Wilks' λ = 0.44, *F*_(4, 30)_ = 9.64, multivariate partial η^2^ = 0.56; ENA: Wilks' λ = 0.28, *F*_(4, 29)_ = 18.6, multivariate partial η^2^ = 0.72; EPA: Wilks' λ = 0.47, *F*_(4, 29)_ = 8.31, multivariate partial η^2^ = 0.53, all *p* < 0.001].

**Table 1:** **Baseline characteristics of the total sample (*N* = 34) of Study 1**.

**Measure**	***M***	***SD***
**DEMOGRAPHICS**		
Age, years	24.0	8.51
Female sex^a^	23	(70)
BMI	21.5	4.73
In a relationship^a^	19	(56)
**BIOBEHAVIORAL VARIABLES**		
Smoke^a^	4	(12)
Smoked units today	0.08	0.28
Cafeine use^a^	29	(85)
Caffeine units today	0.45	1.03
Alcohol use^a^	12	(86)
Alcohol units last 24 h	0.39	1.77
Drug use^a^	4	(12)
Drugs today^a^	0	(0)
Current mental health complaints	2	(6)
Current psychological treatment	3	(9)

*BMI, Body Mass Index*.

a *Indicated with number of positive responses (percentage)*.

**Table 2:** **Mean affect scores at baseline and after every film fragment in Study 1**.

**Phase**	**Implicit**				**Explicit**			
	**NA**		**PA**		**NA**		**PA**	
	***M***	***SD***	***M***	***SD***	***M***	***SD***	***M***	***SD***
Baseline	2.55	0.53	3.20	0.88	1.48	0.67	2.88	0.54
Anger	3.00	1.01	2.51	1.20	2.52^a^	0.90	2.38^a^	0.50
Happy	2.14	0.77	3.70	1.06	1.44	0.59	2.75	0.67
Fear	2.79	0.80	2.67	0.84	2.37	0.75	2.52	0.45
Neutral	2.59	0.81	2.95	1.06	1.45	0.54	2.30	0.61

*N = 34. NA, Negative affect; PA, Positive affect*.

a *N = 33*.

Subsequently, affect after each film clip was compared with baseline through planned comparisons, tested one sided since our hypotheses had a clear direction (e.g., Ludbrook, 2013). The results were corrected for multiple comparisons using the Benjamini-Hochberg procedure with the false discovery rate set at 10% (Simes, 1986; Benjamini and Hochberg, 1995; McDonald, 2014). Results, displayed in Table 3, indicated that compared with baseline (*M* = 2.55, *SD* = 0.53) INA scores were significantly higher after the anger inducing film clip (*M* = 3.00, *SD* = 1.01) and lower after the happiness inducing clip (*M* = 2.14, *SD* = 0.77), *t*_(33)_ = 2.79, *p* = 0.009, *d* = 0.56 and *t*_(33)_ = −3.22, *p* = 0.003, *d* = 0.62, respectively. INA was not significantly different after the fear inducing (*M* = 2.79, *SD* = 0.80) and neutral film (*M* = 2.59, *SD* = 0.81) clips compared with baseline, *t*_(33)_ = 1.59, *p* = 0.122, *d* = 0.35 and *t*_(33)_ = 0.22, *p* = 0.830, *d* = 0.06, respectively. Similarly, compared with baseline (*M* = 3.20, *SD* = 0.88), IPA was significantly lower after the anger inducing clip (*M* = 2.51, *SD* = 1.20), *t*_(33)_ = −2.83, *p* = 0.008, *d* = 0.66 and significantly lower after the fear inducing clip (*M* = 2.67, *SD* = 0.84), *t*_(33)_ = −2.60, *p* = 0.014, *d* = 0.62. IPA was significantly higher after the happiness inducing film clip (*M* = 3.70, *SD* = 1.06), *t*_(33)_ = 2.46, *p* = 0.019, *d* = 0.51. IPA was not significantly changed after the neutral film clip [*M* = 2.95, *SD* = 1.06; *t*_(33)_ = 1.12, *p* = 0.272, *d* = 0.26].

**Table 3:** **Planned comparisons between affect at baseline and after each film clip in Study 1**.

**Comparisons**	***M diff***	***SE***	***t***	***d***
**IMPLICIT NA**				
Anger	0.453	0.16	2.79^**^	0.56
Happy	−0.407	0.13	−3.22^**^	0.62
Fear	0.245	0.15	1.59	0.35
Neutral	0.033	0.15	0.22	0.06
**IMPLICIT PA**				
Anger	−0.691	0.24	−2.83^**^	0.66
Happy	0.495	0.20	2.46^*^	0.51
Fear	−0.534	0.21	−2.60^*^	0.62
Neutral	−0.255	0.23	−1.12	0.26
**EXPLICIT NA**				
Anger^a^	1.027	0.17	5.90^***^	1.31
Happy	−0.032	0.11	−0.30	0.06
Fear	0.891	0.15	5.96^***^	1.25
Neutral	−0.029	0.10	−0.29	0.04
**EXPLICIT PA**				
Anger^a^	−0.521	0.11	−4.92^***^	0.96
Happy	−0.12	0.11	−1.19	0.21
Fear	−0.359	0.09	−4.00^***^	0.72
Neutral	−0.582	0.12	−4.87^***^	1.01

*N = 34. d is calculated with original means and standard deviations. Tests were performed one sided and corrected for multiple comparisons using the Benjamini-Hochberg procedure (Simes, 1986; Benjamini and Hochberg, 1995) with the false discovery rate set at 10%. NA, Negative affect; PA, Positive affect*.

a *N = 33*.

* *p < 0.05*,

** *p < 0.01*,

*** *p < 0.001*.

ENA scores were, compared with baseline (*M* = 1.48, *SD* = 0.67), significantly higher after the anger inducing clip (*M* = 2.52, *SD* = 0.90) and the fear inducing clip (*M* = 2.37, *SD* = 0.75) with *t*_(32)_ = 5.90, *p* < 0.001, *d* = 1.31 and *t*_(33)_ = 5.96, *p* < 0.001, *d* = 1.25, respectively. ENA was not significantly changed after the happiness inducing (*M* = 1.44, *SD* = 0.59) and neutral film clips (*M* = 1.45, *SD* = 0.54), with *t*_(33)_ = −0.30, *p* = 0.767, *d* = 0.06 and *t*_(33)_ = −0.29, *p* = 0.772, *d* = 0.04, respectively. Finally, compared with baseline (*M* = 2.88, *SD* = 0.54), EPA was significantly lower after the anger inducing film clip (*M* = 2.38, *SD* = 0.50), the fear inducing film clip (*M* = 2.52, *SD* = 0.45) and the neutral film clip (*M* = 2.30, *SD* = 0.61) with *t*_(32)_ = −4.92, *p* < 0.001, *d* = 0.96, *t*_(33)_ = −4.00, *p* < 0.001, *d* = 0.72 and *t*_(33)_ = −4.87, *p* < 0.001, *d* = 1.01, respectively. EPA was not significantly changed after the happiness inducing film clip (*M* = 1.75, *SD* = 0.67), *t*_(33)_ = −1.19, *p* = 0.241, *d* = 0.21. Furthermore, there were no significant correlations between changes in implicit affect and explicit affect as displayed in Table 4.

**Table 4:** **Pearsons product-moment correlations between changes in implicit and explicit affect in Study 1**.

**Affect**	**Fragment**	***r***	
		**ENA**	**EPA**
INA	Anger	0.26	0.10
	Happy	0.01	−0.32^+^
	Fear	−0.07	0.11
	Neutral	−0.01	0.33^+^
IPA	Anger	−0.06	0.06
	Happy	−0.06	0.32^+^
	Fear	0.28	−0.21
	Neutral	0.10	−0.34^+^

*N = 34. INA, Implicit negative affect; IPA, Implicit positive affect; ENA, Explicit negative affect, EPA, Explicit positive affect*.

+ *p < 0.10*.

### Discussion

In this study we tested whether the IPANAT is able to detect changes in affective state. The film clips instigated affect-congruent changes on the IPANAT subscales that were unrelated to changes in self-reported affect. These results add to the evidence for the IPANAT's validity by using stimuli that are more “ecologically valid” than the pictures used in the original studies (Quirin et al., 2009a).

Notably, the fear inducing clip lowered IPA, but did not change INA, while the anger evoking clip did change both scales in the expected directions. The fear inducing clip might not have effectively evoked the targeted emotion, anxiety. Still, although not significantly, it did change INA in the expected direction, and yielded expected and significant explicit NA changes. Moreover, in the film clip pool (Schaefer et al., 2010) the same clip yielded a comparable mean ENA of 2.40. Together, this seems to indicate that the negative affect induced by the fear clip was not captured by the INA subscale of the IPANAT. Similarly, although explicit affect changed in an affect-congruent fashion, no changes in EPA were found after the happiness inducing clip. However, considering that EPA did not only decrease after the two negative clips, but also after the neutral film clip, the absence of an affect after the happiness inducing clip can be interpreted as an affect-congruent effect. An alternative explanation could be that the sample had a relatively high positive affect at baseline that did not change after the happiness inducing clip, as it was congruent with the dominant affective state, but did decrease to a relatively more neutral state after the neutral film. Furthermore, one could argue that the differences in length of the film clips elicited different intensities of the induced affect (Gross and Levenson, 1995). However, longer exposure time to a film clip did not increase the effect of the film clips, i.e., the fear inducing film clip was the longest but did not elicit the largest effect.

In sum, the results suggest that the IPANAT is able to measure changes in affect after emotion induction using films that are congruent with the valence of these stimuli. Moreover, it measures changes independently of explicit measures.

## Study 2

### Methods

#### Participants

Thirty three Dutch undergraduate students from Leiden University, The Netherlands were recruited and received eight Euro or course credits for participation. Participants were randomly assigned to the stressor with harassment and stressor without harassment conditions (see below). Two participants had current CV disease and/or psychological problems, in one case the experiment failed due to technical difficulties and one participant had consumed over 5 units of alcohol in the 24 h before the experiment. These cases were excluded from the analysis. The final sample with a mean age of 21.0 (*SD* = 2.29) consisted of 18 females (62.1%). The study was approved by the Independent Ethics Committee of the Institute of Psychology of Leiden University, under number 3145923676.

#### Implicit and explicit affect

The Dutch full version IPANAT was used in this study as a single measure 1 min after the termination of the stressor. The artificial word “safme” was omitted as subjects reported it was associated with “save me,” and thus possibly not sufficiently ambiguous. Leaving out one of the words did not affect reliability; Cronbach's α was 0.93 for INA and 0.92 for IPA, which is in line with previous findings (Quirin et al., 2009a; Brosschot et al., 2014).

As an explicit measure of affect a Visual Analog Scale (VAS) was provided. Participants were asked to what extent they felt a certain emotion (e.g., “How annoyed are you at this moment?”), using the same emotional adjectives as in the IPANAT. At the bottom of the screen a horizontal line of 10 cm was shown, with “not at all” on the left and “very much” on the right on which the participants could indicate their affect, resulting in a score in the range of −100 to +100, with a higher rating indicating increased levels of the adjective. Scores were averaged into ENA and EPA in a similar fashion as the IPANAT. With respect to reliability Cronbach's α's were 0.90 and 0.96 for ENA and EPA, respectively.

#### Cardiovascular activity

The physiological data were measured continuously throughout the experiment. Averages of each outcome measurement were calculated over the last minute of baseline, the 5-min stressor phase and separately for all 15 min of the recovery. Systolic BP (SBP) and diastolic BP (DBP) [in millimeters of mercury (mmHg)] were measured with the Portapres Model-2 (Finapres Medical Systems, Amsterdam, The Netherlands), a non-invasive method to measure BP by placing a finger cuff on the middle finger of the non-dominant hand. The electrocardiogram (ECG) was recorded with Kendall^®;^ 200 Covidien electrodes at a sample rate of 200 Hz with BIOPAC MP150, Biopac Systems, Goleta, CA, USA and visually inspected as well as corrected for movement artifacts with Acqknowledge 3.9.1.4. SBP, DBP and HR (in beats per minute, bpm) were extracted with a tailor made toolbox in Matlab R2012b. A low-pass filter (20 Hz, Blackman 40 coeffients) was applied to the BP signal. The ECG signal was up sampled to 1000 Hz and a comb filter (50 Hz, Q = 5) was applied. Root mean squared successive differences (RMSSD; ms) was derived from the ECG signal as a measure of HRV (Berntson et al., 2007; Nussinovitch et al., 2011; Smith et al., 2013; Munoz et al., 2015). Total peripheral resistance (TPR; in mmHg.min/L) was derived using an approximation of cardiac output (CO) by the formula CO = (0.002*(SBP – DBP))*HR (Sun et al., 2005; Hill et al., 2011, 2012). From MAP and the approximated CO, using the formula TPR = (MAP/CO), estimated TPR was then obtained (Sherwood et al., 1990). To avoid redundancy, only the outcome measure of interest, TPR, is reported.

#### Stress induction

All participants were instructed to perform a mathematical task; calculating backwards from 9000 in steps of 17 out loud. Emotional stress was induced by an anger harassment procedure in the stressor with harassment condition only; participants received seven pre-recorded remarks in an angry tone at set times (0:30; 1:00; 1:30; 2:30; 2:40; 4:00; and 4:55) during the 5 min duration of the stressor phase. These harassing remarks, such as “You are counting too slow, try to speed up.” and “Could you really try to focus now?”, were similar to those used by Radstaak et al. (2011) and others (e.g., Glynn et al., 2002; Mauss et al., 2007). Participants in the stressor without harassment condition did not receive any harassing remarks, but all participants received the instruction to start at 0:00.

#### Procedure

The study was run by two experimenters, of which one monitored the physiological measurements and the other was in contact with the participant. The procedure was explained to the participants after which they signed an informed consent before starting with the experiment. Demographics and biobehavioral variables were obtained followed by placement of the finger cuff and electrodes. The tasks and tests were presented via computer (E-Prime 2.0.8.90). A 5 min baseline period started during which participants could read a magazine with neutral content and were asked to sit quietly (e.g., Gerin, 2011). This was followed by the stress induction as described above. The immediately ensuing recovery started with a minute during which participants did not perform any tasks and were instructed to remain seated for measurement purposes. This was considered to be different from baseline since cognitive representations of the stressor were assumed to be present. After the first minute of recovery the IPANAT started, followed by the VAS. When finished with the tasks within 15 min after the stressor, participants would wait until the 15 min had passed (See Figure 1). Finally, the finger cuff and electrodes were removed and participants were asked about their thoughts and experiences during and about the experiment before they were given a debriefing on the actual purpose of the study and constructs assessed with the IPANAT.

#### Statistical analyses

To represent reactivity, but not recovery, change scores were calculated by subtracting baseline values from those during the stressors for all CV outcomes (Llabre et al., 1991) and effects of condition (i.e., stressor with and without harassment) were analyzed with one sided *t*-tests since our hypotheses had a specific direction (e.g., Ludbrook, 2013). Hierarchical multiple regression was used to assess the association between affect measures and physiological outcome variables, after controlling for condition. Recovery was analyzed with multilevel analyses for SBP and DBP (Lehman et al., 2015), as it has various advantages over repeated measures ANOVAs when analyzing effects of time, such as a better handling of missing data and including individual slopes into the model and thus is able to consider multiple levels in the data (e.g., Llabre et al., 2001). The mean of the CV measure during the stressors was included as covariate in the basic growth model. The model fit did not increase when adding both the baseline and task-related activity and by applying a random slope we already corrected for inter-individual variance unrelated to the stressor (Llabre et al., 2001; Singer and Willett, 2003; Lehman et al., 2015). Grand mean centering was applied to all predictors and covariates. For SBP and DBP separate models were built, but for all models Time was the level 1 variable, representing the measurements' course over 15 min (Model 1). Level 2 represented the person level, which included implicit (Model 2) or explicit affect (Model 3) or both (Model 4). The fit of the models was determined by significant changes in the Akaike information criterion (AIC) and Bayesian information criterion (BIC; Llabre et al., 2001). The data did not allow for multilevel analysis on HR, RMSSD, and TPR as visual inspection showed that recovery of these outcome measures occurred within 1 min after the stressor. Accordingly, for these outcome measures instead of multilevel analyses partial correlations were performed on the first minute of the recovery phase with the affect measures while correcting for CV activity during the stressors. All analyses were done with SPSS 21.0.

### Results

The data were inspected for collection errors, missing values, outliers (>3 *SD*s from the mean) and violation of assumptions for all performed analyses. The distribution of RMSSD was skewed and a square root transformation was applied. One participant displayed a high SBP at rest (>175 mmHg) and throughout the experiment, which was considered extreme. To be conservative, these data points were not included in analyses. Furthermore, one participant provided too many identical responses, i.e., 1-1-1-1 on the IPANAT, and that data was excluded from the data set. As suggested by Quintana and Heathers (2014) differences between conditions regarding demographical and bio-behavioral variables were examined but none were observed, nor were there differences found between conditions in CV outcome measures as displayed in Table 5.

**Table 5:** **Baseline characteristics for the total sample of Study 2 by condition**.

**Measure**	**Harassment (*****n*** = **14)**		**No harassment (*****n*** = **15)**		
	***M***	***SE***	***M***	***SE***	***t/*χ^2^**
**DEMOGRAPHICS**					
Age, years	20.6	0.69	21.3	0.52	−0.73
Female sex^a^	7	(50)	11	(73)	1.68
BMI	21.7	0.91	22.2	1.07	−0.30
In a relationship					
**BIOBEHAVIORAL VARIABLES**					
Smoke^a^	2	(14)	1	(6)	−0.45
Daily Smoking	0.93	0.73	0.60	0.60	0.35
Cafeine use^a^	11	(79)	9	(60)	−1.17
Daily caffeine intake^c^	1.50	0.49	0.90	0.26	1.09
Alcohol use^a^	12	(86)	13	(87)	−0.01
Weekly alcohol consumption	3.09	0.76	2.72	0.97	0.30
Drug use^a^	1	(7)	0	(0)	−1.11
Exercice^a^	11	(79)	13	(87)	−0.33
Weekly exercise (hours)	3.11	0.75	3.37	0.96	−0.21
Visits to GP (last 6 months)	0.79	0.21	1.00	0.45	−0.43
**CARDIOVASCULAR MEASURES**					
SBP^b^	129.2	3.23	124.5	3.55	0.97
DBP	68.3	2.02	68.5	1.95	−0.16
HR	72.2	2.01	79.4	3.27	−1.93^+^
RMSSD^b^	6.14	0.41	5.78	0.35	0.66
TPR^b^	3.17	0.06	3.19	0.10	−0.16

*A square root transformation was applied to RMSSD. There were no significant differences between the conditions. BMI, Body Mass Index; GP, General practitioner; SBP, Systolic Blood Pressure; DBP, Diastolic Blood Pressure; HR, Heart Rate; RMSSD, Root Mean Square of Successive Differences; TPR, Total Peripheral Resistance*.

a *Indicated with number of positive responses (percentage), Pearson χ^2^ was used as test statistic*.

b *N = 28*.

c *Levene's Test indicated unequal variances, df = 19.9*.

+ *p < 0.10, tested two-sided*.

#### Explicit and implicit affect

To examine the effect of the stressor with and without harassment on affect independent samples *t*-tests were performed, one-sided (e.g., Ludbrook, 2013), and corrected for multiple comparisons using the Benjamini-Hochberg procedure with the false discovery rate set at 10% (Simes, 1986; Benjamini and Hochberg, 1995; McDonald, 2014). In response to the stressor higher levels of ENA were reported by participants after the stressor with harassment (*M* = −46.2, *SD* = 37.6) compared with the stressor without harassment [*M* = −74.97, *SD* = 21.89; *t*_(26)_ = 2.47, *p* = 0.020, 95% *CI* [4.83, 52.6], *d* = 0.93]. Furthermore, after the stressor with harassment lower EPA (*M* = −7.60, *SD* = 43.2) was reported compared with the stressor without harassment [*M* = 31.9, *SD* = 36.3; *t*_(27)_ = −2.67, *p* = 0.013, 95% *CI* [−69.9, −9.15], *d* = 0.99]. However, there was no condition effect on INA [with harassment: *M* = 2.97, *SD* = 0.54, without harassment: *M* = 2.97, *SD* = 0.46; *t*_(26)_ = 0.030, *p* = 0.976, 95% *CI* [−0.38, 0.39], *d* = 0.01], nor on IPA [with harassment: *M* = 3.34, *SD* = 0.75, without harassment: *M* = 3.43, *SD* = 0.52; *t*_(26)_ = −0.37, *p* = 0.713, 95% *CI* [−0.59, 0.41], *d* = 0.14]. In sum, there was no condition effect on implicit affect, but there was an expected condition effect on ENA.

As exploratory analyses the associations between the affect measures were examined. INA was not significantly related to IPA or EPA (*r*s < −0.20, *p*s > 0.05) ENA [*r*_(28)_ = 0.16, *p* > 0.05, IPA was not significantly related to ENA [*r*_(28)_ = −0.20, *p* > 0.05], and marginally significantly related to EPA [*r*_(28)_ = 0.32, *p* = 0.09]. ENA and EPA showed a strong inverse relationship [*r*_(28)_ = −0.83, *p* < 0.001].

#### Cardiovascular reactivity

First, we examined whether there were statistically significant changes in CV activity from baseline during both tasks using paired *t*-tests, one-sided (e.g., Ludbrook, 2013), and corrected for multiple comparisons using the Benjamini-Hochberg procedure with the false discovery rate set at 10% (Simes, 1986; Benjamini and Hochberg, 1995; McDonald, 2014). Compared to baseline in both conditions there was an increase in SBP, DBP and HR and a decrease in TPR (see Table 6). No significant decrease was found for RMSSD. Second, we examined the effect of the stressor with and without harassment on the CV measures using independent samples *t*-tests, again one-sided (e.g., Ludbrook, 2013) and with the Benjamini-Hochberg correction. These tests indicated that the stressor with harassment elicited significantly higher SBP (*M* = 23.3, *SD* = 9.43) compared with the stressor without harassment [*M* = 12.6, *SD* = 8.56; *t*_(25)_ = 3.07, *p* = 0.005, 95% *CI* [3.51, 17.8], *d* = 1.19]. DBP was significantly higher in the stressor with harassment (*M* = 12.9, *SD* = 1.40) compared with the stressor without harassment [*M* = 8.98, *SD* = 4.26; *t*_(26)_ = 2.27, *p* = 0.032, 95% *CI* [−2.13, 0.09], *d* = 1.61, respectively]. Furthermore, TPR was significantly lower in the stressor with harassment condition (*M* = −1.44, *SD* = 0.42), compared with the stressor without harassment [*M* = −0.34, *SD* = 0.26; *t*_(18.62)_ = 3.07, *p* = 0.036, 95% *CI* [−2.13, −0.08], *d* = 1.16, respectively]. No significant differences (*p* > 0.10) in HR (*d* = 0.62) and RMSSD (*d* = 0.12) were found between conditions. These findings were confirmed by Repeated Measures ANOVAs. Gender, body mass index (BMI) and smoking were not related to the outcome measures and were not included in the models.

**Table 6:** **Cardiovascular activity during manipulation in Study 2**.

**Measure^b^**	**Total Sample^a^**			**Condition**				
				**Harassment**		**No Harassment**		
	***M***	***SE***	***t***	***M***	***SE***	***M***	***SE***	***t***
SBP	144.1	2.92	−8.75^***^	153.4	4.53	137.2	3.14	−3.07^**^
DBP	78.7	1.71	−11.6^***^	78.9	2.67	77.7	2.31	−2.27^+^
HR	85.2	1.89	−5.75^***^	82.8	3.36	86.8	2.51	−1.63
RMSSD	5.84	0.24	1.14	6.09	0.38	5.63	0.30	0.31
TPR	9.26	0.344	3.48^**^	8.67	0.497	9.74	0.478	2.33^+^

*All tests were performed one sided and corrected for multiple comparisons using the Benjamini-Hochberg procedure with the false discovery rate set at 10%. A square root transformation was applied to RMSSD. SBP, Systolic Blood Pressure; DBP, Diastolic Blood Pressure; HR, Heart Rate; RMSSD, Root Mean Square of Successive Differences; TPR, Total Peripheral Resistance*.

a *Compared with baseline*.

b *Stressor with harassment has two missing values for SBP and RMSSD and one for DBP and HR. Stressor without harassment has one missing value RMSSD and TPR*.

+ *p < 0.10*,

** *p < 0.01*,

*** *p < 0.001*.

#### Cardiovascular reactivity and affect

The association between implicit and explicit affect and CV reactivity was examined with a hierarchical regression analysis for each CV outcome measure resulting in five separate models. In all the models condition was added at step 1 and explicit affect at step 2. Since we expected that implicit affect would explain CV activity over and above explicit affect, we added INA and IPA in step 3. Even though ENA and EPA were highly correlated [*r*_(28)_ = −0.83, *p* < 0.001], VIF and tolerance were of acceptable levels in all tests and thus the assumption of multi-collinearity was not violated (Tabachnick and Fidell, 2007). The final models are displayed in Table 7.

**Table 7:** **Summary of hierarchical multiple regressions for the CV change scores during the stressors in Study 2**.

	**SBP (mmHg)**^a^			**DBP (mmHg)**^b^			**HR (bpm)**^b^			**RMSSD (ms)**^a^^,^ ^c^			**TPR (mmHg.min/L)**^d^		
	***B***	***SE***	**β**	***B***	***SE***	**β**	***B***	***SE***	**β**	***B***	***SE***	**β**	***B***	***SE***	**β**
Constant	-3.57	21.6		-3.57	9.78		-2.98	19.4		4.38^*^	1.82		3.33	1.98	
Condition	−10.0^*^	4.76	−0.49	−3.11	2.28	−0.34	−4.65	4.51	−0.27	0.13	0.48	0.07	−0.61	0.49	−0.24
Explicit NA	−0.09	0.10	−0.30	−0.08	0.05	−0.56	0.07	0.09	0.26	0.005	0.01	0.20	−0.004	0.01	−0.11
Explicit PA	−0.07	0.10	−0.22	−0.05	0.05	−0.49	0.03	0.09	0.16	0.009	0.01	0.39	0.01	0.01	0.38
Implicit NA	8.54^*^	3.89	0.40	2.10	1.86	0.21	6.33	3.68	0.34	−1.02^*^	0.38	−0.52	−1.05^*^	0.40	−0.41
Implicit PA	2.30	3.73	0.14	2.83^+^	1.61	0.38	1.40	3.18	0.10	−0.42	0.35	−0.27	−0.35	0.38	−0.17
*F*		2.60^+^			1.98			1.35			1.79			5.27	
*R*^2^		0.41			0.33			0.25			0.32			0.34	
Δ*R*^2^		0.16			0.15			0.12			0.30^*^			0.20^+^	

*The table shows the associations between condition, affect and CV change scores as generated by the final model (step 3); condition was added at step 1, Explicit NA and PA at step 2 and Implicit NA and PA at step 3 to indicate the additional value of the implicit measure. The F statistic refers to that of the final model. ΔR^2^ is the difference in explained variance between step 2 and step 3 the additional variance of the change scores explained by INA and IPA. NA, negative affect; PA, positive affect; SBP, Systolic Blood Pressure; DBP, Diastolic Blood Pressure; HR, Heart Rate; RMSSD, Root Mean Square of Successive Differences; TPR, Total Peripheral Resistance*.

a *N = 25*.

b *N = 26*.

c *A square root transformation was applied*.

d *N = 24*.

+ *p < 0.10*,

* *p < 0.05*.

SBP was not significantly associated with ENA and EPA. However, INA and IPA were marginal significantly associated and explained an additional 16.1% of the variance [*F*_(5, 19)_ = 2.60, *p* = 0.059, Δ*F* = 2.58, *p* = 0.104]. The final model explained 40.7% of the variance, with condition [*t*_(24)_ = 2.10, *p* = 0.049] and INA [*t*_(24)_ = 2.19, *p* = 0.041] as significant univariate predictors. These results indicate that condition and a high level of INA were associated with an increased SBP. Regarding DBP, ENA and EPA, nor INA and IPA were significantly associated with the outcome measure. However, in the final model IPA was a marginal significant univariate predictor [*t*_(25)_ = 1.76, *p* = 0.093; i.e., higher IPA, higher DBP]. The total variance explained was 33.2%. HR reactivity was not associated with ENA and EPA, nor INA and IPA. Total variance explained, by condition, was 25.2%. For RMSSD, ENA and EPA were not significantly associated. However, although INA and IPA did not significantly affect the model [*F*_(5, 19)_ = 1.79, *p* = 0.16, Δ*R*^2^ = 0.30, Δ*F* = 4.17, *p* = 0.032] INA was a significant univariate predictor in the model *t*_(24)_ = −2.67, *p* = 0.015. The model explained 32.0% of the total variance and indicates thata higher INA was associated with a decrease in RMSSD during the stressor. Finally, reactivity of TPR was significantly associated with ENA and EPA at step 2 and explained 17.8% of the variance compared with step 1, *F*_(3, 20)_ = 4.86, *p* = 0.011, Δ*R*^2^ = 0.18, Δ*F* = 3.08, *p* = 0.07. In the final model INA and IPA showed a significant association [*F*_(5, 18)_ = 5.27, *p* = 0.004, Δ*R*^2^ = 0.172, Δ*F* = 3.82, *p* = 0.041] and explained 58.4% of the total variance. INA was the only significant univariate predictor in the model, [*t*_(23)_ = −2.63, *p* = 0.017]. Again, a higher INA was related to a decrease in TPR during the stressor.

#### Cardiovascular recovery and affect

Multilevel modeling was applied to SBP and DBP. First, a growth model was fitted to the data to model the change over time, Model 1 (Lehman et al., 2015). Second, two separate models for the implicit (Model 2) and explicit (Model 3) scales were fitted that included the affect scales and their interaction with Time and Time^2^, to examine the relation of the affect measures independently. Finally, a model was fitted that included both subscales (Model 4), to examine the hypothesis that implicit affect can explain CV activity over and beyond explicit affect. The models were evaluated with and without condition as a predictor, but adding condition did not improve the models. Models without condition are reported.

**Figure 2:**
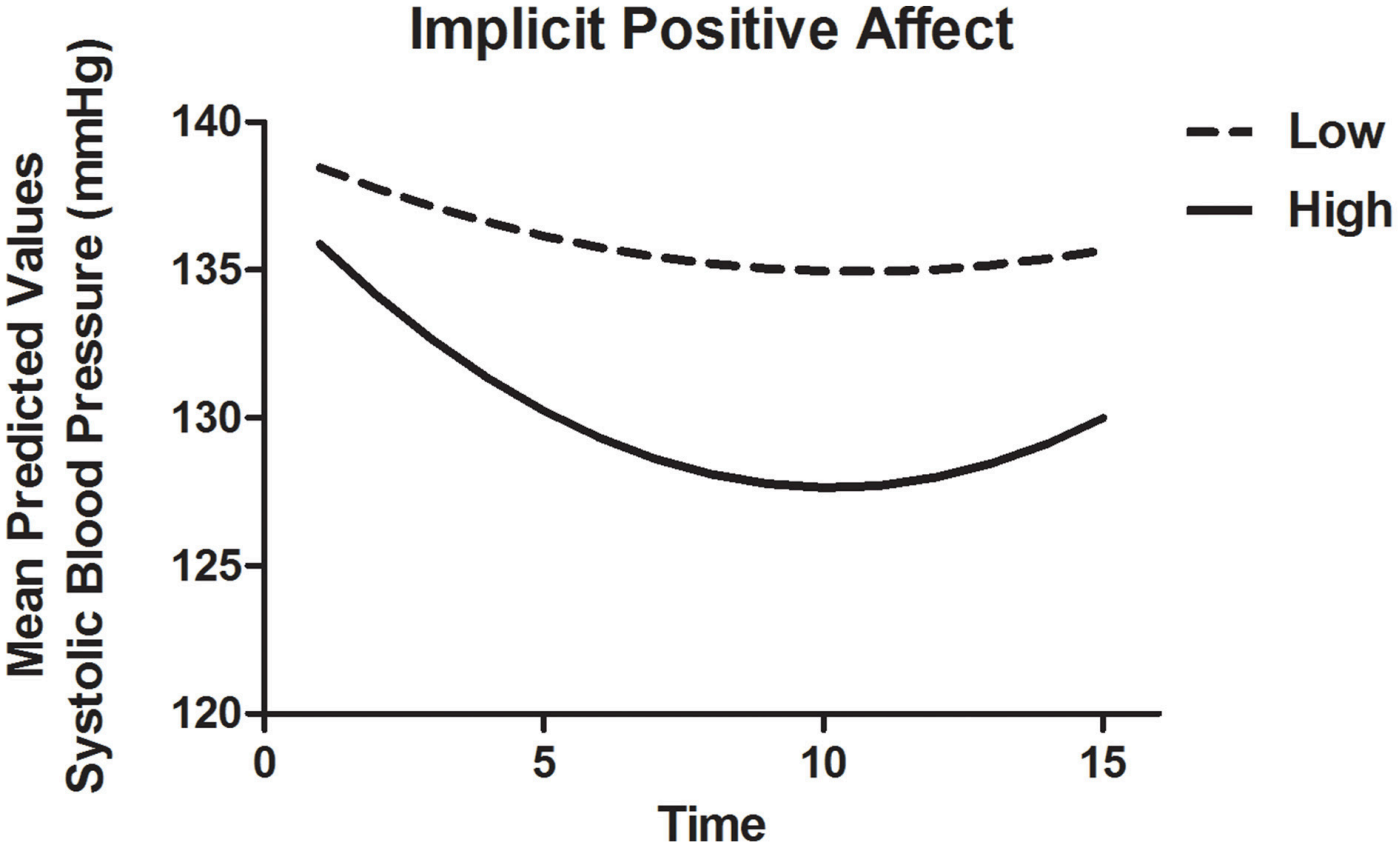
**Mean predictive values of SBP over each of the 15 min of recovery (Model 4) displayed for high and low implicit positive affect**. For display purposes scores of implicit positive affect were dichotomized.

To model SBP recovery, a heterogeneous autoregressive covariance structure was applied to the error variance, as is appropriate for fitting growth models (see for example Singer and Willett, 2003). The slope of Time was allowed to vary randomly between participants. Results are displayed in Table 8A. There were significant associations of Time as well as Time^2^, indicating that the recovery slope was composed of a linear decrease as well as a quadratic change (Model 1). The latter represented a trend with the fastest decrease at the beginning and a (small) increase in SBP toward the end of the recovery phase. Adding INA and IPA and their interactions with Time and Time^2^ (Model 2) improved the model with a Δ*AIC* = 70.8 and Δ*BIC* = 48.1. IPA in interaction with Time and Time^2^ showed marginal significance with *B* = −1.13, *t*_(58.2)_ = −1.94, *p* = 0.057 and *B* = 0.06, *t*_(43.6)_ = 1.90, *p* = 0.098, respectively, indicating that higher IPA was related to a stronger linear decrease of SBP and a stronger quadratic response. Thus, higher IPA was associated with a faster recovery of SBP, especially in the beginning of the recovery phase as displayed in Figure 2. By adding ENA and EPA (without implicit affect) and interactions with Time and Time^2^ (Model 3), the fit also improved with Δ*AIC* = 68.1 and Δ*BIC* = 45.5. However, no individual predictors were found. Additionally, the *AIC* and *BIC* were higher than Model 2, with −2.72 and −2.56, respectively, indicating a better fit of Model 3. When both implicit and explicit affect and interactions with Time and Time^2^ were added to the model (Model 4), it was a better fit to the data compared with Model 1 (Δ*AIC* = 141.1 and Δ*BIC* = 96.2), Model 2 (Δ*AIC* = 70.3 and Δ*BIC* = 48.1) and Model 3 (Δ*AIC* = 73.0 and Δ*BIC* = 50.7). The interactions of IPA and Time [*B* = −1.54, *t*_(55.2)_ = 2.30, *p* = 0.025] and Time^2^ [*B* = 0.08, *t*_(44.2)_ = 2.30, *p* = 0.026] were significantly associated with recovery of SBP in the final model. INA, ENA and EPA were not associated with SBP.

**Table 8A:** **Summary of multilevel analysis for recovery of SBP (mmHg)**.

**Predictor**	**Model 1**			**Model 2**			**Model 3**			**Model 4**		
	***B***	***SE***	***t***	***B***	***SE***	***t***	***B***	***SE***	***t***	***B***	***SE***	***t***
Constant	137.1	1.59	86.2^***^	137.0	1.60	85.6^***^	137.0	1.67	82.0^***^	136.8	1.70	80.4^***^
Time	−1.23	0.35	−3.54^***^	−1.34	0.34	−3.90^***^	−1.20	0.36	−3.37^**^	−1.35	0.35	−3.84^***^
Time^2^	0.07	0.02	3.42^**^	0.07	0.02	3.81^***^	0.06	0.02	3.23^**^	0.07	0.02	3.70^***^
SBP task	0.74	0.08	9.26^***^	0.75	0.09	8.61^***^	0.69	0.08	8.39^***^	0.73	0.09	7.76^***^
Implicit NA				−2.55	3.42	−0.75				−2.76	3.56	−0.78
Implicit PA				0.26	2.66	0.10				1.32	3.24	0.41
Time^*^Implicit NA				0.22	0.73	0.30				0.14	0.74	0.85
Time^*^Implicit PA				−1.13	0.58	−1.84^+^				−1.54	0.67	−2.30^*^
Time^2^^*^Implicit NA				−0.03	0.04	−0.77				−0.03	0.04	−0.73
Time^2^^*^Implicit PA				0.06	0.03	1.90^+^				0.08	0.03	2.30^*^
Explicit NA							−0.06	0.09	−0.70	−0.05	0.09	−0.53
Explicit PA							−0.07	0.07	−0.94	−0.06	0.08	−0.76
Time^*^Explicit NA							0.01	0.02	0.76	0.02	0.02	0.93
Time^*^Explicit PA							0.005	0.02	0.29	0.02	0.02	1.28
Time^2^^*^Explicit NA							−0.0003	0.001	−0.26	−0.0008	0.001	−0.73
Time^2^^*^Explicit PA							−0.0003	0.0009	−0.36	−0.001	0.0009	−1.22
*AIC*		2347.5			2276.7			2279.4			2206.4	
*BIC*		2438.8			2390.7			2393.3			2342.6	
*N*		23			29			29			35	

*Error at Level-1 was organized with a heterogeneous autoregressive first-order covariance structure. At Level-2 the covariance was unstructured. Predictors were grand mean centered. SBP, systolic blood pressure; NA, Negative Affect; PA, Positive affect; N, number of parameters; AIC, Akaike information criterion; BIC, Bayesian information criterion*.

+ *p < 0.10*,

* *p < 0.05*,

** *p < 0.01*,

*** *p < 0.001*.

**Figure 3:**
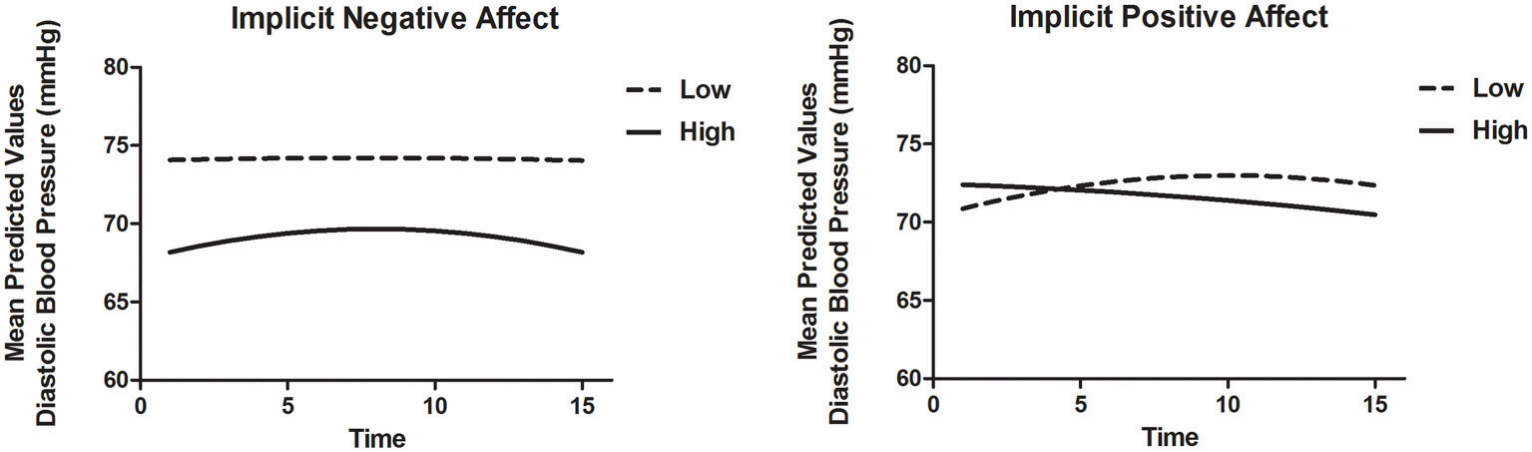
**Mean predictive values of DBP over each of the 15 min of recovery (Model 4) displayed for high and low implicit negative affect and high and low implicit positive affect**. For display purposes scores of implicit affect were dichotomized.

To model DBP recovery, an autoregressive covariance structure was applied to the error variance, as is appropriate for fitting growth models (see for example Singer and Willett, 2003). The slope of Time was allowed to vary randomly between participants. Results are displayed in Table 8B. There was a significant association of Time and Time^2^, indicating that the recovery slope was composed of a linear increase as well as a quadratic change representing an increase at the beginning and an decrease in DBP toward the end of the recovery phase (Model 1). Adding INA and IPA and interactions with Time and Time^2^ (Model 2) improved the model with a Δ*AIC* = 80.0 and ΔBIC = 56.5. Here, INA showed a positive significant interaction with Time [*B* = 0.50, *t*_(89.0)_ = 2.06, *p* = 0.043] and a negative significant interaction with Time^2^ [*B* = −0.04, *t*_(67.4)_ = −2.26, *p* = 0.027]. These associations indicate that higher INA was related to a smaller decrease in DBP with in fact a slight increase at first. Additionally, the IPA by Time interaction was significant with *B* = −0.45, *t*_(89.5)_ = −2.46, *p* = 0.016, indicating that higher IPA was related to a faster linear recovery of DBP over time. Adding EPA and ENA to the model did not substantially improve the model (Model 3). When both implicit and explicit affect and interactions with Time and Time^2^ were added to the model (Model 4), the fit did not improve and the associations between implicit affect and DBP recovery remained. The results are illustrated in Figure 3. Separate models of both SBP and DBP were also run with gender, BMI and smoking as covariates. Adding these covariates to the models did not change the associations of implicit and explicit affect with SBP and DBP recovery.

**Table 8B:** **Summary of multilevel analysis for recovery of DBP (mmHg)**.

**Predictor**	**Model 1**			**Model 2**			**Model 3**			**Model 4**		
	***B***	***SE***	***t***	***B***	***SE***	***t***	***B***	***SE***	***t***	***B***	***SE***	***t***
Constant	71.5	0.62	115.8^***^	71.3	0.56	126.0^***^	71.6	0.61	116.5^***^	71.3	0.58	122.8^***^
Time	0.25	0.12	2.46^*^	0.22	0.11	1.99^*^	0.29	0.11	2.55^*^	0.26	0.11	2.38^*^
Time^2^	−0.02	0.008	−2.46^**^	−0.02	0.007	−2.30^*^	−0.02	0.007	−2.70^**^	−0.02	0.007	−2.60^*^
DBP Task	0.85	0.07	13.06^***^	0.94	0.07	14.1^***^	0.81	0.07	12.3^***^	0.91	0.07	12.5^***^
Implicit NA				0.69	1.24	−0.56				−0.76	1.27	−0.59
Implicit PA				−0.81	0.94	−0.86				−0.30	1.09	−0.28
Time^*^Implicit NA				0.50	0.24	2.06^*^				0.59	0.24	2.45^*^
Time^*^Implicit PA				−0.45	0.18	−2.46^*^				−0.41	0.19	−2.15^*^
Time^2^^*^Implicit NA				−0.04	0.02	−2.26^*^				−0.04	0.02	−2.66^**^
Time^2^^*^Implicit PA				0.01	0.01	1.21				0.02	0.01	1.22
Explicit NA							−0.04	0.03	−1.18	−0.02	0.03	−1.09
Explicit PA							−0.04	0.03	−0.04	−0.02	0.03	−0.85
Time^*^Explicit NA							−0.006	0.006	−0.93	−0.007	0.006	−1.09
Time^*^Explicit PA							−0.005	0.005	−1.12	−0.004	0.005	−0.75
Time^2^^*^Explicit NA							0.0003	0.0004	0.73	0.0004	0.0004	1.11
Time^2^^*^Explicit PA							0.0003	0.0003	0.41	0.001	0.0003	0.40
*AIC*		1732.7			1652.7			1669.6			1593.5	
*BIC*		1768.7			1712.3			1729.1			1676.0	
*N*		9			15			15			21	

*Error at Level-1 was organized with an autoregressive first-order covariance structure. At Level-2 the covariance was unstructured. Predictors were grand mean centered. DBP, diastolic blood pressure; NA, Negative Affect; PA, Positive affect; N, number of parameters; AIC, Akaike information criterion; BIC, Bayesian information criterion*.

* *p < 0.05*,

** *p < 0.01*,

*** *p < 0.001*.

As mentioned before recovery of the other outcome measures took place within 1 min after the stressors had ended and could therefore not be modeled over time using multilevel analysis. Alternatively, to test the association with the affect measures partial correlations were performed on the first minute after recovery of the means of HR, RMSSD and TPR, correcting for the preceding reactivity. HR, RMSSD and TPR were not significantly related to implicit or explicit affect. Results are displayed in Table 9.

**Table 9:** **Pearson product-moment partial correlations between measures of affect and first minute of recovery of Study 2**.

**Affect**	**HR^a^**	**RMSSD^b^**	**TPR^b^**
Implicit NA	−0.24	0.30	0.17
Implicit PA	−0.20	−0.14	0.25
Explicit NA	−0.18	−0.001	0.18
Explicit PA	0.16	−0.05	−23

*Controlled for HR, RMSSD and TPR during the stressor. A square root transformation was applied to RMSSD. There were no significant correlations. NA, Negative affect; PA, Positive affect; HR, Heart Rate; RMSSD, Root Mean Square of Successive Differences; TPR, Total Peripheral Resistance*.

a *N = 23*.

b *N = 22*.

### Discussion

Study 2 examined whether affect measured at an implicit level, as measured with the IPANAT, was associated with CV reactivity to and CV recovery after a stressor with or without anger harassment. During both stressors participants showed increased SBP, DBP and HR, and lower TPR compared with baseline. When comparing the two conditions, these associations were more pronounced for SBP, DBP and TPR after the stressor with harassment compared with the stressor without harassment. HR and RMSSD responses were similar for both conditions. Taken together this suggests a more pronounced cardiac controlled vascular response during harassment in addition to a math stressor.

There were no differences between the conditions in implicit affect. In contrast, those in the stressor with harassment condition experienced more ENA and less EPA as expected. This indicates that the more negative affective component of the harassment stressor was only reflected in explicit affect and not in implicit affect. However, higher INA was related to higher SBP reactivity and lower RMSSD and TPR reactivity during the stressors independent of stressor type. No associations between implicit affect and DBP and HR levels were observed during the stressors. Unexpectedly, the pattern of recovery was similar for both conditions. Overall, BP recovered rather slowly after an initial somewhat faster decrease. Importantly, the slow recovery of BP over the course of the recovery was (partly) statistically explained by implicit affect, but not by explicit affect. More precisely, slow recovery of SBP was related to low IPA, but not to INA. Slow recovery of DBP was partly related to both high INA and low IPA. HR, RMSSD, and TPR seem to have recovered rather quickly, that is, within the first minute after the stressor. For these outcome measures no relationship with implicit affect measures was found. Remarkably, explicit affect was not related to any of the CV measures.

Taken together, the most salient result of Study 2 seems to be that not explicit, but implicit affect explained variance in reactivity and recovery, but that at the same time explicit, but not implicit affect, was influenced by the stressor types, and thus by the experimental increase in negative emotionality. One explanation of these contrasting results might be that self-reported (explicit) affect reflected mainly the experimental demand characteristic (“the experimenter made me angry so I think I am angry”) while implicit affect reflected the core affective state induced by both stressors (Russell, 2003), which was not substantially influenced by the harassment, as will be discussed below.

## General discussion

Traditional self-report measurements of stress, or explicit measures of affect, cannot fully explain CV activity. Hence, the relationship between affect as an indicator of psychological stress and CV health remains largely indeterminate, and the examination of a possible role for implicit measures of affect is warranted. In the present work the IPANAT, as a promising implicit measure of affect, was evaluated in two studies to examine its ability to assess changes in affective state and explain stress-related CV activity beyond explicit measures of affect. In Study 1 the IPANAT appeared to be able to measure affect-congruent changes in INA and IPA after anger and happiness inducing film clips. Of the multiple expected congruent effects only an effect on IPA, but not INA, after a fear inducing clip was found. Importantly, implicit affect changed independently from explicit affect. Thus, the IPANAT is able to measure changes in affect that are generally congruent with the valence of the presented stimuli and independent of explicit affect. We conclude that the differential responses of the IPANAT in response to the film clips form an important extension of the modest number of available validation studies of the IPANAT and add ecological validity to previously used methods (e.g., pictorial stimuli).

Study 2 employed a realistic stressor with and without an enhanced negative affective component and continuously measured CV activity. The affective component was reflected in differences in explicit affect, but not in implicit affect. Nevertheless, only the implicit affect measures, and not the explicit ones, were associated with the CV responses to both stressors and their recovery afterwards. Specifically, SBP increases and HRV and TPR decreases during the stressors were related to higher INA, but implicit affect did not clearly relate to DBP and HR reactivity. Slower recovery of SBP was associated with lower levels of IPA, and DBP recovery was associated with both IPA and INA in the expected direction. HR, HRV and TPR showed a very quick recovery that was not related to implicit or explicit affect. Thus, the IPANAT adds to the understanding of the CV response to stressors were explicit measure do not. These results and some unexpected findings, such as the prolonged physiological effects of the stressors on BP but not HR, HRV or TPR, are discussed in more detail below.

### Stressors and CV activity

We did not find a direct effect of the manipulation of the stressors on recovery, but the differences in recovery can be attributed to the differences in reactivity. The stressors yielded higher SBP and DBP and lower TPR, and for all CV measures the magnitude of reactivity contributed to speed of recovery. This suggests a role for the reactivity, not the stressor itself, in the effect of a stressor on the speed of CV recovery. Consequently, the notion of Brosschot et al. (2014) and Linden et al. (1997) that an emotional stressor would delay CV recovery compared with non-emotional stressor holds to the extent that it increases reactivity that, independent from condition, slows down recovery.

In general, the pattern of CV activity in Study 2, a vascular (i.e., BP) and myocardial (i.e., HR) increase during the stressor and a prolonged recovery that appeared to be mostly vascular under cardiac control, is comparable to other studies (e.g., Haynes et al., 1991; Gregg et al., 1999; Glynn et al., 2002; Mauss et al., 2007; Juster et al., 2012; Brindle et al., 2014). The quick recovery of HR is in line with the observation that an increase in HR can be seen as primarily reflecting task engagement or effort (e.g., Berntson et al., 1996), and less related to possible emotional aspects of the task that might linger on after its completion. Furthermore, the speech activity required in the current stress task (i.e., calculating loudly) might also have played a role. Sloan et al. (1991) found a smaller increase in HR during a mathematical task when vocalization of the response was not required. More specifically, changes in respiratory frequency due to speaking were found to increase HR. The neccesity to speak ended right after the task resulting in a quick decrease of HR. Sloan et al. (1991) also attributed the absence of changes in HRV to the effect of speaking on HRV. Thus, the findings regarding HR and HRV might not or to a lesser extent be related to the psychological component of the stressors but rather to the design characteristcs of the study.

In contrast to what is commonly found in threatening situations, namely an increase in TPR, we here found a decrease in TPR (Blascovich, 2008; Seery, 2011). It is possible that the stressors, a mathematical task with or without harassment, did not induce a threatened but a challenged state. Regarding our findings with TPR the stressors might not have been as straining as we had anticipated, for example because of lack of personal relevance of the stressors to the participants (Blascovich, 2008). The findings also suggests that the prolonged effects on SBP and DBP cannot be explained by TPR, that recovered within a minute after the stressor, but are due to other factors that we have not measured directly, such as stroke volume or cardiac output. Overall, the results support previous notions that researchers should include recovery in the laboratory models of stress, as the activity seen during reactivity does not necessarily reflect clinically relevant responses (Linden et al., 1997).

### IPANAT and CV activity

The findings of Study 1 add to the understanding of affect, measured at an implicit and explicit level, by addressing the ongoing nature of affect through presentation of film clips. Furthermore, the absence of changes in INA after the fear inducing clip is similar to the study of Quirin et al. (2011) in which they showed a threat-related film clip and measured INA and IPA but found no changes in implicit affect after a threat inducing film clip. This suggests that the INA subscale might not be sensitive or specific enough to detect fear. The construct validity, both convergent and discriminant, seem supported by Study 1: the scores on the IPANAT scales are reasonably congruent with the emotional content of the different emotional film clips. This was only partially the case for Study 2 where only convergent validity seems apparent from the expected correlations with physiological measurements stress responses. In line with previous research, we observed no association between INA and IPA, which explains why the results we found with INA did not always mirror those with IPA (Quirin et al., 2009a,b).

The stressors in Study 2 led to group specific changes in explicit but not implicit affect. This is even more surprising considering the independent relation we found between implicit affect and CV outcome measures. The increased ENA and decreased EPA can be explained by demand characteristics of the stressors. In the condition with harassment the affective component was quite obvious to the participants. They were told they were not doing a good job. In the stressor group without harassment there was no feedback which created an ambiguous setting. These differences might very well be what was measured with the explicit measures of affect; the ambiguous situation was not experienced as overtly negative. An alternative explanation is that in Study 2 that the IPANAT scores were in fact related to the trait component, and not the state component, of affect (Quirin et al., 2009a). As no baseline measure of the IPANAT was taken, the current study does not exclude this possibility; perhaps it is the trait part of affect captured by the IPANAT that is related to CV activity. However, it is likely that self-reported affect reflected what the participants thought they had to report and not necessarily how they were feeling, i.e., their core affect (Russell, 2003). Moreover, core affect might be best reflected on the IPANAT subscales; both stressors elicited discomfort which was overridden by demand characteristics of the experiment on the explicit level of affect but was displayed in both conditions on the implicit level. This explanation is further amplified by the finding that only implicitly measured affect contributed to CV activity during and after the stressors. If this interpretation is correct, implicit affect scores reflected core affect that was manifested in CV changes. This highlights the additional value of implicit measures, or the IPANAT in particular, in addressing the relation between stress and CV diseases (Egloff and Schmukle, 2002; Egloff et al., 2002; Verkuil et al., 2014).

The role of positive affect in the development of disease has not been explicitly addressed in the unconscious perseverative cognition hypothesis, which emphasizes the health consequences of stress-related cognition beyond awareness (e.g., Brosschot et al., 2010). However, in the current study we found that a higher IPA is related to higher DBP reactivity and lower IPA is related to slow recovery of both SBP and DBP. This is consistent with the results of Quirin et al. (2009b, Study 1) who found that increased IPA, not INA, measured during 2 days, was related to a lower cortisol awakening response and total diurnal cortisol the following day in addition to EPA. The finding that IPA is related to CV activity and cortisol excretion provides new insights in the relation between the IPANAT and two biological mechanisms.

Overall, the prolonged BP responses were best explained by implicit affect more than any other variable measured. Together these results suggest that stress-related cognition beyond self-report is related to physiological effects of stress, but, importantly, reduced levels of IPA play an equally detrimental role.

### Limitations

The results should be interpreted while considering some limitations. In Study 2 the sample sizes, particularly regarding the two conditions, were rather small which increases the risk for Type 2 error, i.e., the study may have been underpowered to reveal statistical significant findings. In this light we have interpreted marginal statistically significant findings in both studies as potentially relevant, which was supported by the effect sizes. Furthermore, in Study 2 there was no neutral condition, merely a mathematical task with and without anger harassment. No differences between conditions were found for affect measured at an implicit level and CV recovery. Adding a true neutral condition without a stressor might provide additional information about the ability of the IPANAT to detect INA induced by a psychological stressor and enabling inferences about the role of affect, measured implicitly and explicitly, in physiological recovery. Alternatively other methods of stress induction could be considered, such as a public speech stressor or the Trier Social Stress Test, which combines a public speech with the anger harassment used in Study 2 (e.g., Kirschbaum et al., 1993). Also, we cannot exclude the possibility that participants differed, despite randomization, in natural math-related abilities, which could have been a confounder. Finally, Study 1 and 2 did not use the same explicit measures and can therefore not be readily compared; it cannot be excluded, for example, that we would have found associations of explicit affect with CV activity in Study 2 if we had used the PANAS used in Study 1. To further clarify the relation between implicit stress-related cognition and CV health, future studies should not be limited to implicit measures of affect after experimentally induced stress, but should also apply the measures to daily life (Mossink et al., 2015) and/or in individuals with chronic stress. Finally, the current experiments focused on the assessment of implicit affect with the IPANAT. However, other measures of implicit constructs to assess other aspects of unconscious stress-related cognition, e.g., action tendencies or emotion recognition, could also provide more information to clarify the relation between psychological stress and CV health.

## Conclusion

The IPANAT is the first specific measure of implicit affect. The current two studies suggest that it is able to measure differences not only between affective responses to pictorial stimuli, as reported previously, but also between fear (with its positive subscale), anger and happiness as elicited using film clips (Study 1). The findings suggest that the IPANAT is associated with CV activity during and after a stressor (Study 2). Importantly, all findings for the IPANAT were independent of those for explicit affect, which were mostly absent.

Notwithstanding the remaining questions and limitations, these findings offer support for the theory that stress beyond self-report measures, i.e., unconscious stress-related cognition, at least partly relates to CV responses, that, when prolonged in daily life, are related to the progress and development of CV diseases. Especially because of this relevance for health, further research is needed to clarify the explanatory value of the IPANAT and possible other implicit measures of stress-related cognition, and their applicability to stress research.

## Author contributions

All authors listed, have made substantial, direct and intellectual contribution to the work, and approved it for publication.

## Funding

This work was supported by a grant from ZON-MW (Netherlands Organization for Health Research and Development; TOP Grant) to JB, nr. 40-00812-98-11029 and a grant from the Netherlands Organization for Scientific Research (NWO) awarded to BV (Veni Grant 451-14-013).

### Conflict of interest statement

The authors declare that the research was conducted in the absence of any commercial or financial relationships that could be construed as a potential conflict of interest.
